# Emotional enhancement of error detection—The role of perceptual processing and inhibition monitoring in failed auditory stop trials

**DOI:** 10.3758/s13415-017-0546-4

**Published:** 2017-10-26

**Authors:** Magdalena Senderecka

**Affiliations:** Cognitive Science Unit, Institute of Philosophy, Jagiellonian University, Grodzka 52, 31-044 Kraków, Poland

**Keywords:** Emotion, Response inhibition, Error monitoring, Stop-signal task, Event-related potentials (ERPs)

## Abstract

The first aim of the present study was to test whether arousing, aversive sounds can influence inhibitory task performance and lead to increased error monitoring relative to a neutral task condition. The second aim was to examine whether the enhancement of error monitoring in an affective context (if present) could be predicted from stop-signal-related brain activity. Participants performed an emotional stop-signal task that required response inhibition to aversive and neutral auditory stimuli. The behavioral data revealed that unpleasant sounds facilitated inhibitory processing by decreasing the stop-signal reaction time and increasing the inhibitory rate relative to neutral tones. Aversive sounds evoked larger N1, P3, and Pe components, indicating improvements in perceptual processing, inhibition, and conscious error monitoring. A first regression analysis, conducted regardless of the category of the stop signal, revealed that both selected indexes of stop-signal-related brain activity—the N1 and P3 amplitudes recorded in the unsuccessfully inhibited trials—significantly accounted for the Pe component variance, explaining a large amount of the observed variation (66%). A second regression model, focused on difference measures (emotional minus neutral), revealed that the affective increase in the P3 amplitude on failed stop trials was the only factor that significantly accounted for the emotional enhancement effect in the Pe amplitude. This suggests that, in general (regardless of stop-signal condition), error processing is stronger if the erroneous response directly follows the stimulus, which was effectively processed on both the perceptual and action-monitoring levels. However, only inhibition-monitoring evidence accounts for the emotional increase in conscious error detection.

Error monitoring is usually defined as the ability to detect and evaluate an error, which may lead to remedial actions. The cerebral basis underlying error monitoring can be investigated by recording event-related potentials (ERPs) from the scalp. With regard to ERPs that have been linked to incorrect motor responses, two components have been studied, namely the ERN (error-related negativity; Gehring, Goss, Coles, Meyer, & Donchin, 1993), also called Ne (error negativity; Falkenstein, Hohnsbein, Hoormann, & Blanke, 1990), and Pe (error positivity; Falkenstein, Hohnsbein, Hoormann, & Blanke, 1991). The ERN is a sharp negative wave, peaking at around 50–100 ms after the onset of an erroneous reaction, that is distributed over the anterior regions (Falkenstein et al., 1990; Gehring et al., 1993). Various theories implicate the ERN as a reflection of the mechanism that monitors the difference between an intended and an actually performed action (Coles, Scheffers, & Holroyd, 2001; Falkenstein et al., 1991), as a signal of reinforcement learning (Holroyd & Coles, 2002), or as a reflection of conflict between simultaneously active correct and incorrect response tendencies (Botvinick, Braver, Barch, Carter, & Cohen, 2001; Yeung, Botvinick, & Cohen, 2004). Most recently it has been considered to reflect the increase in attentional control, involving enhanced activation within the medial frontal cortex, typically observed in situations demanding ongoing monitoring of performance (van Noordt, Campopiano, & Segalowitz, 2016; van Noordt, Desjardins, Gogo, Tekok-Kilic, & Segalowitz, 2017; van Noordt, Desjardins, & Segalowitz, 2015). In addition, the ERN has also been proposed to reflect the subjective significance of an error (Gehring et al., 1993; Hajcak, Moser, Yeung, & Simons, 2005) or the accompanying negative affect, which signals the need for remediation and control (Hajcak & Foti, 2008; Inzlicht & Al-Khindi, 2012; Schmeichel & Inzlicht, 2013).

The ERN is followed by a sustained Pe component that exhibits a more posterior and central scalp distribution (Falkenstein et al., 1991). It has been considered to reflect the conscious stage of error detection (Nieuwenhuis, Ridderinkhof, Blom, Band, & Kok, 2001), affective processing of erroneous response (Falkenstein, 2004), a P3-like component related to the motivational significance of an error (Leuthold & Sommer, 1999; Ridderinkhof, Ramautar, & Wijnen, 2009), or the accumulation of evidence that an error has occurred (Steinhauser & Yeung, 2010; see also Ullsperger, Harsay, Wessel, & Ridderinkhof, 2010; Wessel, Danielmeier, & Ullsperger, 2011).

There is good evidence that long-lasting negative affect associated with psychiatric diseases or character traits goes along with enhanced error detection. Increased performance monitoring has been observed in patients suffering from major depression (Chiu & Deldin, 2007; Holmes & Pizzagalli, 2008), as well as in participants who are worried, emotionally distressed (such as patients with obsessive–compulsive disorder) or experiencing high negative affect (Gehring, Himle, & Nisenson, 2000; Hajcak, McDonald, & Simons, 2003; Johannes et al., 2001; Luu, Collins, & Tucker, 2000). However, relatively few ERP studies have examined the influence of short-duration affective states, induced by emotional stimuli, on error monitoring. Larson and colleagues observed that pleasant pictures superimposed on flanker stimuli enhanced the ERN amplitude relative to neutral or unpleasant pictures (Larson, Perlstein, Stigge-Kaufman, Kelly, & Dotson, 2006). In turn, Wiswede and colleagues noticed that unpleasant pictures presented 700 ms prior to flanker stimuli increased the size of the ERN relative to neutral or pleasant pictures (Wiswede, Münte, Goschke, & Russeler, 2009). In both studies, task-irrelevant emotional stimuli were used to induce an affective state. In addition, an enhanced ERN was observed in studies that used more abstract emotional manipulation to examine whether error monitoring is sensitive to the motivational impact of punishment or to the induction of feelings of helplessness (Pfabigan et al., 2013; Riesel, Weinberg, Endrass, Kathmann, & Hajcak, 2012). However, some studies have also failed to observe ERN amplitude variation in response to fear or sad and happy mood induction (Moser, Hajcak, & Simons, 2005; Olvet & Hajcak, 2012; Paul, Walentowska, Bakic, Dondaine, & Pourtois, 2017). Moreover, Ogawa and colleagues have shown that verbal admonishment following erroneous responses decreased the ERN relative to the no-feedback condition (Ogawa, Masaki, Yamazaki, & Sommer, 2011). Importantly, in the majority of these studies the analyses were limited to the first component of the ERN-Pe error-related complex. However, scattered evidence suggests that short-duration affective states induction may also influence Pe amplitude (Moser et al., 2005; Paul et al., 2017).

Recently, Senderecka (2016) investigated the influence of emotional, task-relevant, visual stimuli on both error-related components simultaneously in a stop-signal paradigm. Participants performed an emotional stop-signal task (SST) that required response inhibition to briefly presented threatening and neutral visual stimuli. The analyses revealed that negative, arousing pictures improved behavioral performance by decreasing the stop-signal reaction time and increasing the inhibitory rate. The ERN amplitude was similar in the emotional and neutral conditions. However, the most interesting and novel finding of the above-mentioned study was that the Pe component, associated with conscious evaluation or affective processing of an error, was significantly enhanced in the negative trials as compared to the neutral trials. It was assumed that the greater Pe amplitude in the negative condition was probably associated with an increase in the significance of an error committed after the presentation of the threatening stop signals, which were more effectively processed on the perceptual and cognitive control levels than the neutral ones. Nevertheless, this assumption was not directly tested in the study.

The present study was designed to expand on Senderecka (2016) by further exploring the mechanism of the emotional enhancement effect on error monitoring. The first goal of the present study was to test whether the previous pattern of results could be obtained with emotional stimuli from a non-visual sensory modality. To reach this goal, a stop-signal task requiring response inhibition to aversive and neutral auditory stimuli was used. Sounds can clearly prompt strong emotional responses, as was shown in a large behavioral study by Cox (2008). However, as compared to visual stimuli, they are still investigated only rarely (Gerdes, Wieser, & Alpers, 2014). The results of electrophysiological studies suggest that aversive auditory stimuli (such as scraping), as compared to neutral sounds, are accompanied by a more pronounced early negativity of event-related brain potentials as a measure of enhanced allocation of attention (Czigler, Cox, Gyimesi, & Horváth, 2007), a finding similar to what has been observed with reactions to emotional pictures (Schupp, Junghöfer, Weike, & Hamm, 2003). This attentional advantage of emotional stimuli appears to be mediated by the amygdala (Anderson & Phelps, 2001). A number of studies have shown that the amygdala processes auditory stimuli and exhibits higher activation in response to unpleasant sounds (or for both unpleasant and pleasant) than in response to neutral sounds (Aubé, Angulo-Perkins, Peretz, Concha, & Armony, 2015; Klinge, Röder, & Büchel, 2010; Kumar, von Kriegstein, Friston, & Griffiths, 2012; Mirz, Gjedde, Sødkilde-Jrgensen, & Pedersen, 2000; Zald & Pardo, 2002). In sum, there is considerable evidence that emotional sounds can serve as a useful research tool to elicit emotions and investigate emotion processing.

The second goal of the study was to test whether emotional enhancement of error monitoring (if present) could be predicted from stop-signal-related brain activity, even if it occurred several hundred milliseconds before error commission. The evidence from error awareness experiments indicates that primary task performance does influence error-related components (for a review, see Wessel, 2012). According to the accumulating evidence model (Ullsperger et al., 2010), information about the accuracy of an action is available from multiple different cortical processors (linked to the sensory, motor, performance monitoring and interoceptive systems) that work in parallel and code different types of evidence. The strength of this evidence accumulates over time and contributes to the detection of an error in a feed-forward fashion. Steinhauser and Yeung (2010) demonstrated that this accumulating evidence is indeed reflected in the amplitude of the Pe component. This observation implies that it should be possible to use the Pe to track the internal processes leading to error detection in the emotional and neutral context, and to predict differences between these two conditions in participants’ error signaling, on the basis of the stop-signal-related brain activity observed in the unsuccessfully inhibited trials. Such an analysis can provide important knowledge about the functionality of performance monitoring in an affective context.

Affective stimuli, in comparison to other events, are better encoded due to the prioritized perceptual processing (Pessoa, 2009; Pessoa, Kastner, & Ungerleider, 2002; Pourtois, Schettino, & Vuilleumier, 2013; Vuilleumier, 2005). When relevant to the task, they can attract further attention and improve inhibitory performance monitoring (Chiu, Holmes, & Pizzagalli, 2008; Pawliczek et al., 2013; Pessoa, Padmala, Kenzer, & Bauer, 2012). Thus, during inhibitory task performance, emotions can impact both lower-order and higher-order cognitive functions. In unsuccessfully inhibited emotional trials, accumulated information from different sensory and executive processors can probably lead to enhanced error monitoring, reflected in the amplitude of the Pe (Senderecka, 2016). This raises the question of which of the stop-signal-related processes is responsible for the emotional enhancement of error detection: increased perceptual processing, more effective inhibitory performance monitoring, or both.

To assess the role of stop-signal-related states in the emotional enhancement of error detection, two components previously studied in the SST in response to the stop signal were used, namely the N1, which is associated with perceptual processing, and P3, which is linked to response inhibition. The auditory N1 is a sustained negativity, peaking over the fronto-central or central regions, which can begin at 60–80 ms and last until 160 ms after the onset of a sound (Näätänen & Picton, 1987; Woods, 1995). It originates mainly in the auditory cortex, reflects the initial extraction of information from the sensory analysis of a stimulus and is very sensitive to selective attention (Hillyard, Hink, Schwent, & Picton, 1973). In the SST context, it was also implicated as a marker of attention already reflecting an inhibitory mechanism (Kenemans, 2015). The P3 component, which peaks around 300–350 ms, resembles the classical P3b with more central distribution (Kok, Ramautar, De Ruiter, Band, & Ridderinkhof, 2004). It has been considered an index of a late stage of monitoring the outcome of the inhibitory process (e.g., Nieuwenhuis, Yeung, van den Wildenberg, & Ridderinkhof, 2003) and, most recently, a reflection of the suppression and slowing of motor behavior or just motor inhibition (Enriquez-Geppert, Konrad, Pantev, & Huster, 2010; Huster et al., 2011).

The present study’s hypothesis was that aversive sounds would induce transient negative emotional processes, which would dynamically modulate behavioral performance by decreasing the stop-signal reaction time and increasing the inhibitory rate. It was also predicted that stop-signal-locked ERP components related to perceptual processing and inhibition monitoring, as well as the error-monitoring response-locked Pe component, would show increased amplitude during an emotional condition. On the basis of previous results (Senderecka, 2016), emotional enhancement of the ERN amplitude was not expected. Finally, it was assumed that emotional enhancement of the Pe component could be predicted from stop-signal-related ERP indexes of perceptual processing and/or inhibition monitoring.

## Method

### Participants

Thirty-seven self-declared right-handed students (28 females and nine males), 20–25 years old (*M* = 21.5 years, *SD* = 1.8), participated in the present study. All participants were in good health, free of medications and had normal or corrected-to-normal vision. None reported a history of psychiatric or neurological diseases. From the initial sample recruited for the study, three participants were excluded because of the small number of successfully inhibited responses (below 20%) in at least one experimental condition—neutral or emotional. Two others were excluded because of excessive sweating, eye blinks and/or muscle artifacts, resulting in an insufficient number of trials to analyze ERPs. The final sample consisted of 32 participants (25 females and seven males). The sample size was determined in accordance with proposed guidelines (Simmons, Nelson, & Simonsohn, 2011). The power analysis performed was based on the study by Pessoa et al. (2012), in which the emotionality of the stop signal had a significant effect on inhibitory performance. The results indicated that a sample size of 32 would allow detection of a medium effect size with a power >80%, at an alpha level of .05.

### Procedure and task

The experimental procedure was in accordance with the ethical principles of the 1964 Declaration of Helsinki (World Medical Organization, 1996). Participants were seated in a dimly lit, sound-attenuated, air-conditioned testing room. After providing written informed consent to take part in the study, all participants completed two emotional stop-signal tasks (one with auditory and the other with visual stop stimuli), with the order of the tasks randomized across participants; only data from the stop-signal task with auditory stop stimuli are presented here. Participants were asked to restrict body movements and blinking as much as possible during the recording of the EEG.

The emotional stop-signal task with auditory stop stimuli required participants to perform a primary binary-choice (or go) response task. It included two visual go stimuli, consisting of an image of a white arrow pointing left or right (picture size: 94 × 61 pixels). These stimuli were presented randomly one at a time, for 100 ms, each with a 50% probability, on a black background in the center of a 23-in. computer monitor, 1 m in front of the participant, at eye level.

Participants were instructed to respond by pressing the left or right “ctrl” key, located on a computer keyboard, according to the direction of the arrow that was presented to them. If the arrow pointed to the left, they were to respond by pressing the left “ctrl” key using their left index finger; if the arrow pointed to the right, they were to respond by pressing the right “ctrl” key using their right index finger. In addition, they were instructed to react to the go stimuli as fast and as accurately as possible. Each trial began with a white central fixation cross (picture size: 30 × 30 pixels) for 800 ms, followed by the picture of an arrow.

In a random sample of 25% of the trials, an emotionally negative (aversive) or neutral sound was presented for 100 ms, which acted as the stop signal. The aversive stimuli consisted of five negative, unpleasant, arousing noises, such as scrunching, scraping or thumping, which do not require a long presentation in order to elicit an emotional response. The neutral stimuli consisted of five simple tones (600, 800, 1000, 1200, and 1400 Hz). The aversive and neutral sounds were adjusted to be equally loud. The peak amplitudes were comparable across the sound categories. The stimuli were presented at 60 dB binaurally through headphones (Sennheiser HD 429).

In a related study, 86 female and 32 male students (mean age 20.6, *SD* = 1.9) rated the valence and arousal levels of the same ten sounds using a previously described procedure (Yang et al., 2014). The valence rating instruction was “Rate how unpleasant or pleasant the sound makes you feel on a scale ranging from 1 to 9 (1 = *very unpleasant*, 5 = *neutral*, 9 = *very pleasant*).” The arousal rating instruction was: “Rate how calm or aroused the sound makes you feel on a scale ranging from 1 to 9 (1 = *calm*, 5 = *somewhat aroused*, 9 = *extremely aroused*).” The results of *t* tests revealed significant differences in both valence, *t*(117) = 6.37, *p* < .001, *d* = 0.7, and arousal, *t*(117) = −8.35, *p* < .001, *d* = 0.7, between the emotional and neutral sounds. The valence ratings were lower for emotional (*M* = 2.9, *SD* = 1.2) than for neutral (*M* = 3.9, *SD* = 1.6) sounds. The students gave higher arousal ratings to emotional (*M* = 6.5, *SD* = 1.3) than to neutral (*M* = 5.3, *SD* = 1.8) stimuli.

**Fig. 1:**
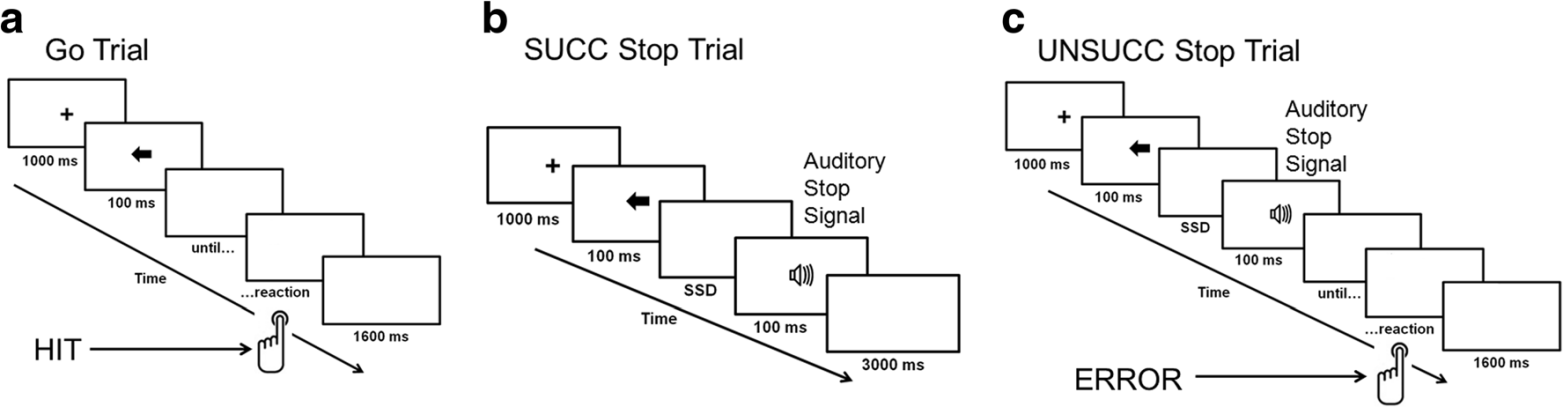
Behavioral task. **a** Go trial, without stop-signal presentation. **b** Successfully inhibited stop-signal trial. **c** Unsuccessfully inhibited stop-signal trial. ERROR = unsuccessfully inhibited response, HIT = correct response to go stimuli, SSD = stop-signal delay, SUCC = successful stop trial, UNSUCC = unsuccessful stop trial.

Aversive and neutral stimuli prompted the participants to inhibit their responses to the primary go task, regardless of which arrow was presented. Each stop signal occurred an equal number of times for each arrow (five times for the left and five times for the right). The interval between the presentation of the go stimulus and the aversive/neutral stop signal was varied trial-by-trial using a tracking method. The interval (i.e., the stop-signal delay, SSD) increased or decreased by 50 ms (from 100 to 400 ms) for the next stop-signal trial, depending on whether the participants successfully inhibited or failed to inhibit their response to the go stimulus. Thus, seven SSDs were possible: 100, 150, 200, 250, 300, 350, and 400 ms. After a successful inhibition, the inter-stimulus interval became longer; after an unsuccessful inhibition, it became shorter. The initial value of the SSD was set to 150 ms. The staircasing was common for two conditions. The presentation of the aversive and neutral stimuli in the stop-signal trials was semirandomly determined, with the restriction that all possible sequences of exposition (aversive followed by aversive, neutral followed by neutral, aversive followed by neutral, neutral followed by aversive) were equally represented in the task (25% for each sequence). The aim of the tracking method was to converge on an SSD where participants successfully inhibited responses in approximately 50% of the stop-signal trials. Figure 1 presents an outline of the stop-signal task design.

Participants received one or two practice blocks of 24 trials before data collection to ensure that they understood the task. After practice runs, they completed eight experimental blocks, each consisting of 50 trials, with short breaks between blocks. The task was implemented using DMDX software (Forster & Forster, 2003) and presented on an Eizo Foris FS2333-BK LCD monitor (60-Hz refresh rate), which offers an excellent image construction time (7.5 ms, on average).

### Electrophysiological recording

The continuous scalp electroencephalogram (EEG) was recorded from 32 silver/silver-chloride (Ag/AgCl) active electrodes (with preamplifiers) using the BioSemi Active-Two system: Fp1/Fp2, AF3/AF4, F3/F4, F7/F8, FC1/FC2, FC5/FC6, T7/T8, C3/C4, CP1/CP2, CP5/CP6, P3/P4, P7/P8, PO3/PO4, O1/O2, Fz, Cz, Pz, and Oz. The electrodes were secured in an elastic cap (Electro-Cap), according to the extended 10–20 international electrode placement system. The zero-reference principal voltage values (each site quantified relative to the driven right leg and common mode sense loop) were digitized at a sampling rate of 256 Hz. The horizontal and vertical electro-oculograms (EOGs) were monitored using four additional electrodes placed above and below the right eye and in the external canthi of both eyes. The electrical signal was not filtered during EEG acquisition. All channels were re-referenced offline to the average of the two mastoid electrodes. The recordings were filtered off-line with a high-pass filter of 0.05 Hz (slope 24 dB/oct) and a low-pass filter of 25 Hz (slope 12 dB/oct). Ocular and other stationary artifacts were removed with the independent component analysis (ICA) algorithm using the Brain Vision Analyzer 2 (Brain Products, Munich, Germany).

### Data quantification

Stimulus-locked (−100 to 700 ms around the stop-signal onset) and response-locked (−150 to 600 ms relative to the key press) segments were subsequently checked and averaged. Contaminated trials exceeding maximum/minimum amplitudes of ± 65 *μ*V were rejected by a semi-automatic procedure. The mean number of rejected trials was low (6% on average). Stop-signal ERPs were averaged separately for each type of stop-signal trial: successful (SUCC) and unsuccessful (UNSUCC), and for each stop-signal condition: emotional (EMO) and neutral (NEU).

In the SST, the ERPs elicited in response to the go and stop stimuli overlap in time, due to the short interval between these two kinds of events. To minimize the possibility of a differential overlap distortion problem across two stop-signal conditions (EMO and NEU), one single staircase for two different stimulus types was used in this study. The analyses confirmed that the SSD directly preceding emotional (*M* = 164.9 ms, *SD* = 43.1) and neutral (*M* = 162.8 ms, *SD* = 40.9) sounds did not differ significantly between these two conditions, *t*(31) = 1.07, *p* = n.s. However, to better control for the potential differential overlap distortion problem, ERP subaverages for the successful EMO, successful NEU, unsuccessful EMO and unsuccessful NEU stop-signal trials were obtained separately for each of the stop-signal delays (from two to six for each participant, depending on individual tracking method results). Then, for each condition, all stop-signal delay subaverages were collapsed together in an equally weighted way, respectively, thereby better equating the overlap from the go stimuli on the stop-signal ERPs (see Pliszka, Liotti, & Woldorff, 2000; Schmajuk, Liotti, Busse, & Woldorff, 2006; Shen, Tsai, & Duann, 2011, for similar procedures). The mean number of correct, artifact-free, epochs included in the ERP analysis across all participants for each of the stop-signal trial categories were as follows: unsuccessful NEU *M* = 29.3 (*SD* = 3.3), unsuccessful EMO *M* = 23.0 (*SD* = 4.3), successful NEU *M* = 17.7 (*SD* = 3.9), successful EMO *M* = 23.6 (*SD* = 4.2).

Motor reaction ERPs were calculated separately for correct (response hit) and unsuccessfully inhibited (response error) responses. In addition, grand averages for incorrect responses were calculated separately for incorrect responses following emotional (EMO response error) and neutral (NEU response error) stop-signal presentations. The mean number of correct, artifact-free, epochs included in the ERP analysis across all participants for each of the response trial categories were as follows: response-hit *M* = 280.3 (*SD* = 20.4); EMO response-error *M* = 23.0 (*SD* = 4.3); NEU response-error *M* = 29.3 (*SD* = 3.3).

After inspection of the grand-average waveforms and scalp topography distributions for each trial type and various difference waves, time windows were selected around N1 (120–190 ms) and P3 (270–400 ms)—locked to the stop-signal presentation, and ERN (0–80 ms) and Pe (120–270 ms)—locked to the motor reaction. Mean voltage amplitudes in the component-specific windows were used for statistical analysis. Stop-signal ERPs were aligned to the pre-stimulus baseline from − 100 to 0 ms, whereas motor reaction ERPs were baseline-corrected relative to the pre-response interval from − 150 to − 50 ms.

### Statistical analyses

To compare inhibitory performance across two stop-signal conditions (emotional and neutral), a series of *t* tests were performed on the behavioral variables—stop-signal reaction time (SSRT) and inhibition rate. Two separate mean SSDs for each condition were obtained by selectively averaging the SSDs, which directly followed in the staircase procedure the presentation of the aversive or neutral stop signal, regardless of the category of the following stop signal. The global SSD was also calculated.

The SSRT, which provides an estimate of the latency of the inhibitory process, was calculated following the procedure of Logan (1994). Reaction times from go stimuli responses in which no stop signal occurred were collapsed into a single distribution and rank ordered. The *n*th reaction time was selected, where *n* was obtained by multiplying the number of no-signal reaction times in the distribution (300) by the probability of responding (e.g., .5 if the global inhibition rate was equal to 50%) for each participant separately. The global SSRT was calculated by subtracting the average SSD from the *n*th reaction time, following the horse race model (see Logan & Cowan, 1984; Verbruggen & Logan, 2008, for more detail). In turn, the SSRTs for each stop-signal condition were calculated by subtracting the emotional/neutral SSD from the *n*th reaction time, chosen on the basis of condition-wise probability of responding.

To analyze the amplitudes of the N1 and P3, two-way repeated measures analysis of variance (ANOVA) was conducted (separately for each component), with the factors being trial type (SUCC vs. UNSUCC) and stop-signal condition (EMO vs. NEU). In turn, to analyze the amplitudes of the ERN and Pe, two one-way repeated measures ANOVAs were conducted (again separately for each component), the first ANOVA with the factor being trial type (response hit vs. response error), and the second ANOVA with the factor being response-error type (EMO response error vs. NEU response error). The use of two-way repeated measures ANOVA was impossible in the case of the ERN and Pe, because emotional manipulation was restricted to the stop-signal and erroneous response trials only. Thus, response hit condition (correct response to go stimuli) was represented by only one (not repeated) measure.

On the basis of the topographical distribution of the grand-averaged ERP activity and according to the literature, different electrode clusters were selected for these components (see Näätänen & Picton, 1987, for the auditory N1 literature review; Kok et al., 2004, for the inhibitory P3; and Overbeek, Nieuwenhuis, & Ridderinkhof, 2005, for the components of error processing). The N1 component was analyzed at the averaged central sites (FC1, FC2, C3, C4, Cz, CP1, and CP2), P3 and Pe were analyzed at the centro-parietal sites (Cz, CP1, CP2, P3, P4, and Pz) and ERN was analyzed at the averaged fronto-central sites (F3, F4, Fz, FC1, FC2, C3, C4, and Cz).

Because one of the main objectives of this experiment was to test which of the stop-signal-related processes is responsible for the emotional enhancement of error detection, multiple regression analyses were performed across individuals to determine whether unique variance in the Pe amplitude could be predicted on the basis of the brain activity that occurred at various stages of unsuccessfully inhibited trials. Continuous variables were examined with the Kolmogorov–Smirnov test and were not statistically different from the normal distribution. The critical *p* value was set at .05 for all the analyses. To interpret significant findings, global analyses were followed by restricted post-hoc *t* tests, with *p* value corrected for multiple comparisons (Bonferroni).

## Results

### Behavioral data

The mean RT of the correct go trials was 373.3 ms (*SD* = 43.7) and the mean go error rate was 1.6% (*SD* = 1.9). The global inhibition rate was slightly below 50% (*M* = 43.8%, *SD* = 6.5), which may suggest that participants were generally more focused on the primary go task than on the secondary stop task. The percentage of successfully inhibited responses differed significantly between the two stop-signal conditions: *M* = 37.3% (*SD* = 7.7) for the neutral condition versus *M* = 50.2% (*SD* = 7.8) for the emotional condition, *t*(31) = 8.60, *p* < .001, *d* = 1.3, indicating that stop performance was higher in the emotional than in the neutral stop-signal trials. This finding suggests that emotional stop signals had a greater capacity than neutral stop signals to withdraw attention from the primary go task. The SSD was significantly longer in trials directly following in the staircase procedure the presentation of an emotional (*M* = 170.2 ms, *SD* = 44.0) rather than a neutral (*M* = 156.2 ms, *SD* = 38.3) stop signal, *t*(31) = 6.10, *p* < .001, *d* = 0.3. Consequently, the SSRT was significantly shorter in the emotional condition (*M* = 203.3 ms, *SD* = 23.3) than in the neutral condition (*M* = 217.3 ms, *SD* = 23.3), which indicates that participants were better at inhibiting the responses with emotional stop signals than neutral stop signals, *t*(31) = 6.10, *p* < .001, *d* = 0.6. The global SSD (also including the first stop-signal delay) was 163.7 ms (*SD* = 41.7), whereas the global SSRT was 209.4 ms (*SD* = 22.6).

### ERP findings

The results of the global analysis conducted on all of the components are presented in Table 1. The mean amplitudes and standard deviations for all components and experimental conditions are shown in Table 2.

**Table 1:** Results of the global analysis of the ERP components

Amplitude				
Effect		*F*	*p*	*η*_p_^2^
Trial Type (SUCC vs. UNSUCC) × Stop-Signal Condition (EMO vs. NEU)				
N1		5.20	=.03	.14
P3		10.08	<.01	.25
Trial Type (SUCC vs. UNSUCC)				
N1		21.85	<.001	.41
P3		20.22	<.001	.40
Response Type (HIT vs. ERROR)				
ERN		42.03	<.001	.58
Pe		71.81	<.001	.70
Stop-Signal Condition (EMO vs. NEU)				
N1		8.28	=.01	.21
P3		39.55	<.001	.56
Error Response Condition (EMO vs. NEU)				
ERN		1.20	=.28	.04
Pe		41.19	<.001	.57

*EMO* emotional stop-signal trials, *ERROR* unsuccessfully inhibited responses, *HIT* correct responses to go stimuli, *NEU* neutral stop-signal trials, *n.s.* not significant, *SUCC* successful stop trials, *UNSUCC* unsuccessful stop trials. *df* = 1,31

**Table 2:** Components’ amplitude results (*μ*V) in all experimental conditions

Component	Mean Amplitude (*SD*)							
Stop-Signal-Locked	UNSUCC	SUCC	NEU	EMO	NEU UNSUCC	EMO UNSUCC	NEU SUCC	EMO SUCC
N1	− 2.8 (5.8)	− 5.6 (6.3)	− 3.2 (5.4)	− 5.2 (6.8)	− 1.3 (5.4)	− 4.3 (7.0)	− 5.1 (5.9)	− 6.1 (7.4)
P3	16.8 (6.4)	20.4 (7.5)	16.2 (6.8)	20.9 (7.0)	13.6 (6.9)	20.0 (6.9)	18.8 (8.0)	21.9 (7.9)
Response-Locked	ERROR	HIT			NEU ERROR	EMO ERROR		
ERN	− 4.4 (5.5)	1.3 (3.8)			− 4.6 (5.7)	− 4.1 (5.8)		
Pe	8.7 (5.4)	− 1.4 (5.4)			6.4 (6.0)	11.0 (5.6)		

*EMO* emotional stop-signal trials, *ERROR* unsuccessfully inhibited responses, *HIT* correct responses to go stimuli, *NEU* neutral stop-signal trials, *SD* standard deviation, *SUCC* successful stop trials, *UNSUCC* unsuccessful stop trials

#### ERPs time-locked to the stop-signal presentation

**Fig. 2:**
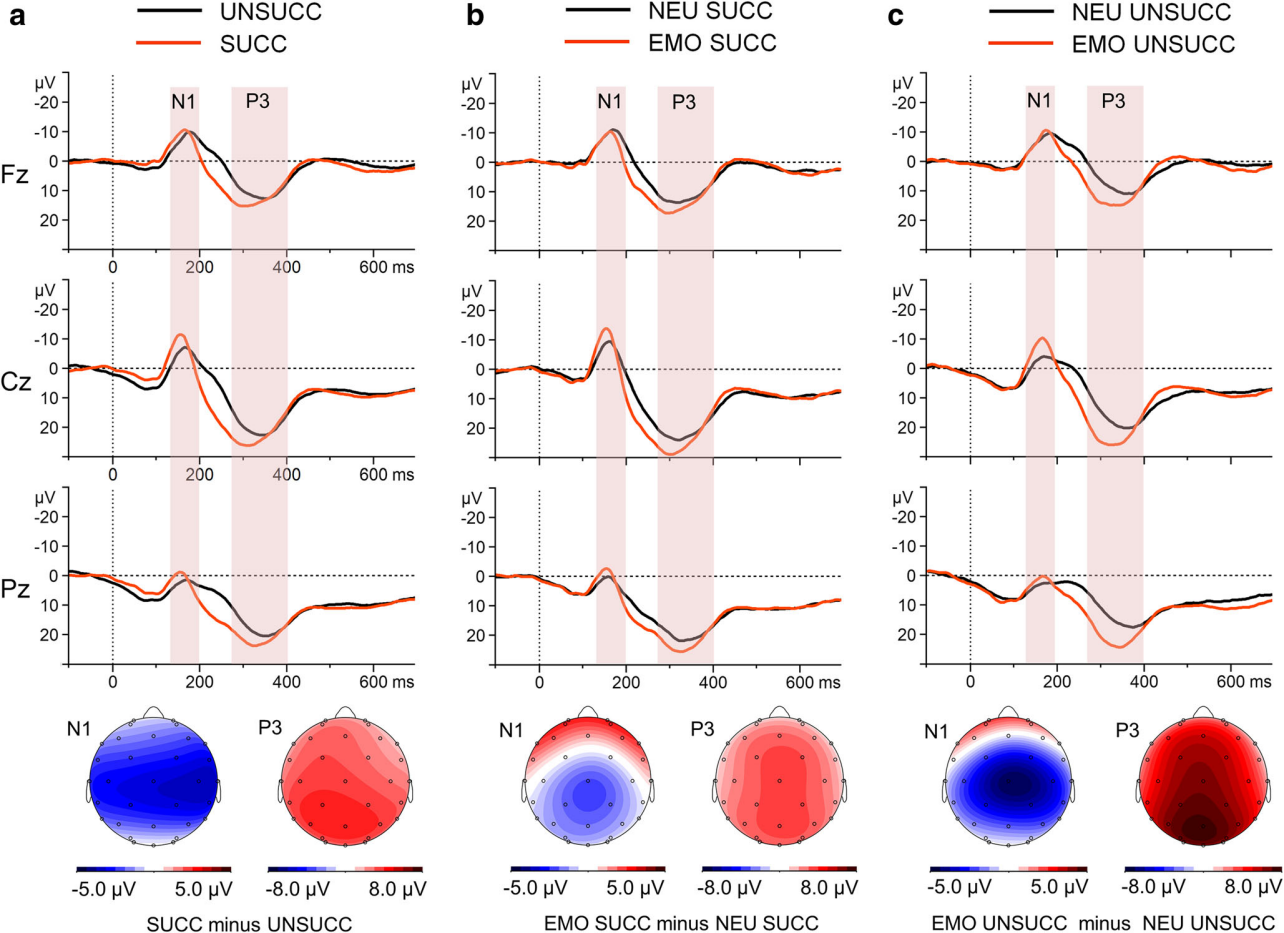
Stop-signal-locked grand-average waveforms at the representative midline electrodes Fz, Cz, and Pz (upper part), with scalp potential difference maps for the N1 and P3 components (bottom part). **a** Grand-average ERPs for successfully and unsuccessfully inhibited trials and topographic maps for the SUCC-minus-UNSUCC difference wave. **b** Grand-average ERPs to the emotional and neutral stop signals in successfully inhibited trials, and topographic maps for the EMO SUCC minus NEU SUCC difference wave. **c** Grand-average ERPs to the emotional and neutral stop signals in unsuccessfully inhibited trials, and topographic maps for the EMO UNSUCC minus NEU UNSUCC difference wave. The component-specific windows examined in this study are highlighted. EMO = emotional stop-signal trials, NEU = neutral stop-signal trials, SUCC = successful stop trials, UNSUCC = unsuccessful stop trials, 0 = time point of stop-signal onset.

Figure 2 presents the grand-average ERPs to the stop signal at representative sites, with scalp distribution maps for the difference waves.

##### N1 component (120–190 ms)

The N1 amplitudes showed a main effect of trial type, in which N1 amplitudes were more pronounced in SUCC than in UNSUCC trials (Δ*M* = 2.8 *μ*V). Emotional stop signals elicited larger N1 amplitudes than neutral stop signals (Δ*M* = − 2.0 *μ*V). In addition, a Trial Type × Stop-Signal Condition interaction was observed. To explain this interaction, post hoc *t* tests were carried out. Although N1 amplitudes were larger for emotional than for neutral sounds in both the SUCC and the UNSUCC trials, the difference reached significance only in the UNSUCC trials, *t*(31) = 3.47, *p* < .01, *d* = 0.5.

##### P3 component (270–400 ms)

Both main effects were significant in the global analysis conducted for the P3 amplitudes—trial type and stop-signal condition. The P3 amplitude was larger in the SUCC trials than in the UNSUCC trials (Δ*M* = 3.6 *μ*V). The P3 amplitude was also more pronounced in the EMO trials than in the NEU trials (Δ*M* = 4.7 *μ*V). In addition, a Trial Type × Stop-Signal Condition interaction effect was observed. Although P3 amplitudes were larger for emotional than for neutral sounds in both the SUCC, *t*(31) = 3.29, *p* < .01, *d* = 0.2, and the UNSUCC, *t*(31) = 7.02, *p* < .001, *d* = 0.8, trials, the size of the effect of the stop-signal condition was larger for the UNSUCC (Δ*M* = 6.4 *μ*V) than for the SUCC (Δ*M* = 3.1 *μ*V) trials.

#### ERPs time-locked to the motor reaction

**Fig. 3:**
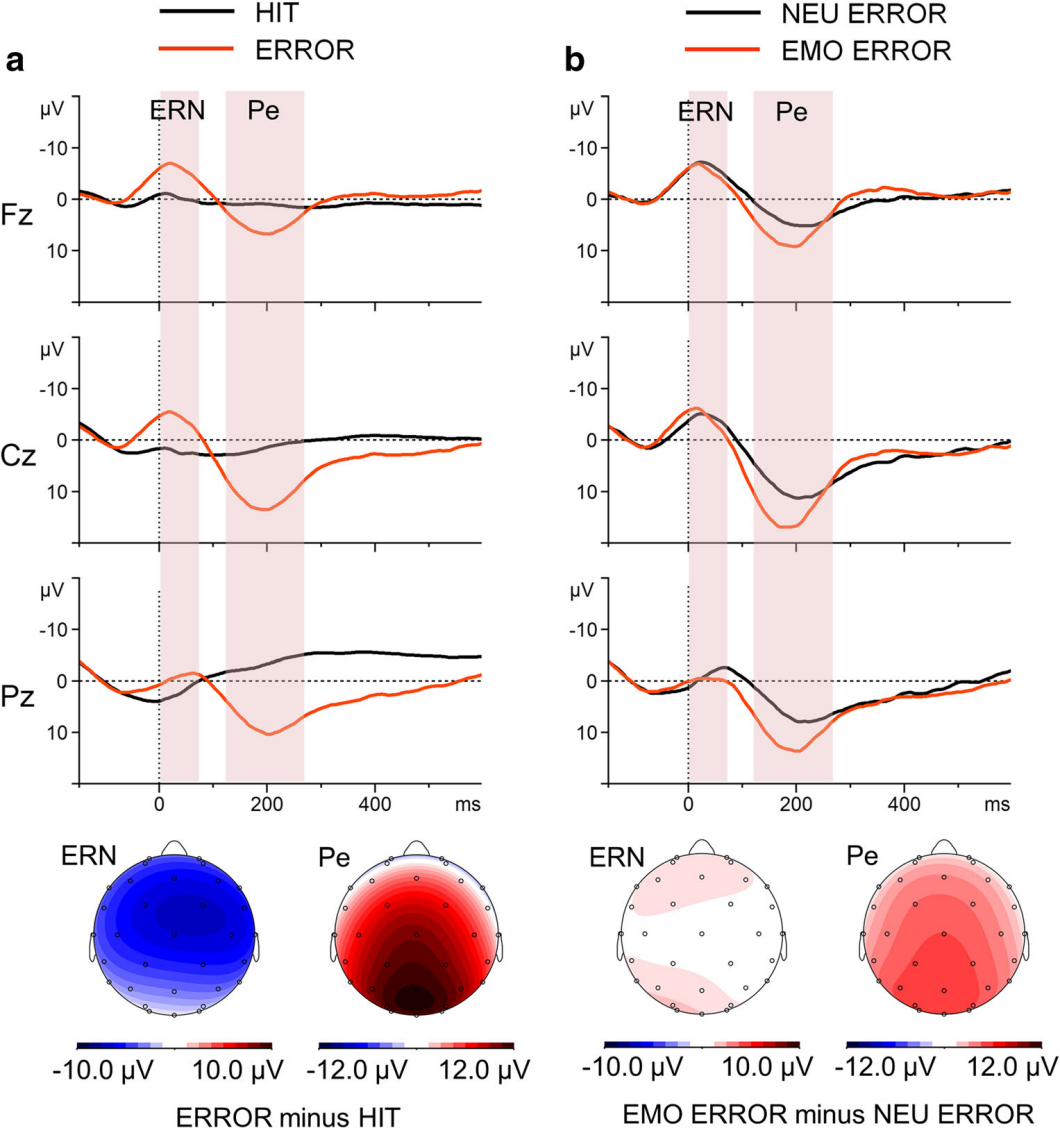
Response-locked grand-average waveforms at the representative midline electrodes Fz, Cz, and Pz (upper part), with scalp potential difference maps for the ERN and Pe components (bottom part). **a** Grand-average ERPs for correct- and erroneous-response trials and topographic maps for the HIT-minus-ERROR difference wave. **b** Grand-average ERPs to erroneous emotional and erroneous neutral responses and topographic maps for the EMO ERROR-minus-NEU ERROR difference wave. The component-specific windows examined in this study are highlighted. EMO = emotional stop-signal trials, ERROR = unsuccessfully inhibited responses, HIT = correct responses to go stimuli, NEU = neutral stop- signal trials, 0 = time point of stop-signal onset.

Figure 3 presents the grand-average ERPs for motor reactions at representative sites, with scalp distribution maps for the difference waves.

##### ERN component (0–80 ms)

The global analysis revealed that the main effect of response type was significant. The ERPs to response errors (UNSUCC trials, time-locked to the button press) showed a sharp negative peak, which was attenuated in the ERPs to response hits (Δ*M* = 5.7 *μ*V). The ERN amplitudes were statistically comparable in the EMO and NEU response error trials (Δ*M* = 0.5 *μ*V).

##### Pe component (120–270 ms)

The ERPs to response errors showed sustained positive activity (following the ERN), which was absent in the ERPs to response hits (Δ*M* = 10.1 *μ*V). Statistical analysis revealed that the main effect of error response condition was significant. The Pe amplitudes time-locked to the motor reaction were greater in the EMO than in the NEU response error trials (Δ*M* = 4.6 *μ*V).

### Exploratory regression analyses

**Fig. 4:**
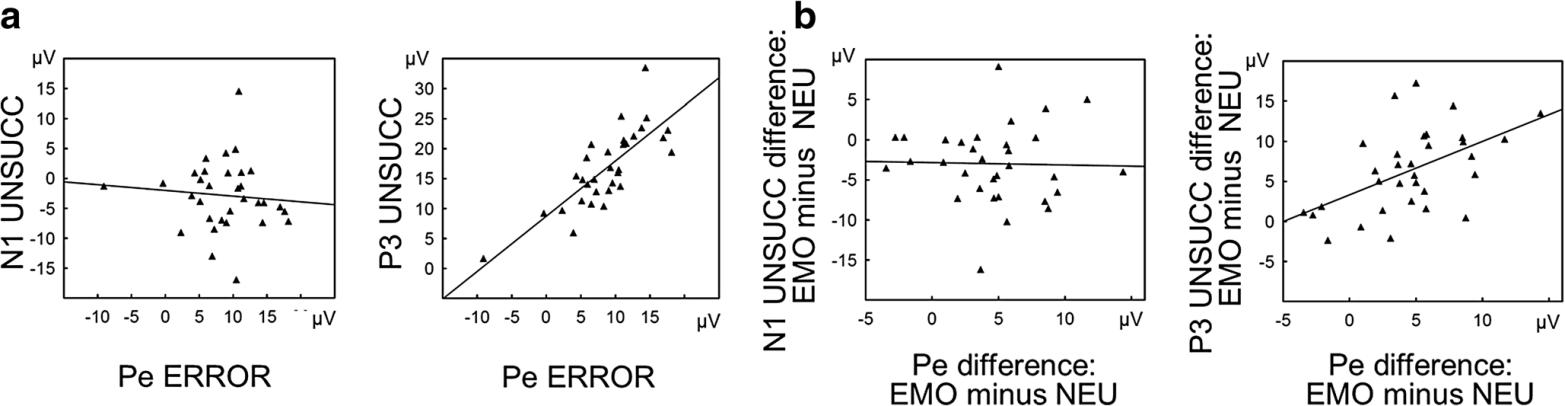
Scatterplots and linear regression lines. **a** Relationships between the Pe amplitude in erroneous-response trials and the amplitude of two stop-signal-locked components, the N1 (left part) and P3 (right part), in unsuccessfully inhibited trials. **b** Relationships between the effect of task condition on the Pe (i.e., the difference in Pe amplitudes between the emotional and neutral task conditions) and the effects of task condition on the N1 (left part) and P3 (right part) (i.e., the differences in the N1 and P3 amplitudes between the emotional and neutral stop-signal conditions). EMO = emotional stop-signal trials, ERROR = unsuccessfully inhibited responses, NEU = neutral stop signal trials, UNSUCC = unsuccessful stop trials.

To further explore associations between the two ERP components time-locked to the stop signal (N1, P3) and the Pe component time-locked to the erroneous motor reaction, for which emotional enhancement effects were observed, two exploratory multiple regression analyses were performed. The first analysis was intended to check whether the Pe component amplitude could be predicted from the N1 and P3 components in the unsuccessfully inhibited trials, regardless of the category of the stop signal. The predictor variables were the N1 and P3 amplitudes in the UNSUCC trials. The Pearson correlation analyses revealed that they were not significantly correlated (*r* = .16, *p* = n.s.). The predicted variable was the Pe amplitude in response error trials. The first regression model explained 66% of the variance in the Pe amplitudes (*R* ^2^ = .66), *F*(2, 29) = 28.56, *p* < .001. The P3 amplitude was clearly the factor that accounted for the largest portion of variance (*ß* = .82), *t* = 7.51, *p* < .001, followed by the N1 amplitude (*ß* = − .22), *t* = −2.05, *p* < .05. Scatterplots and linear regression lines from the first analysis are illustrated in Fig. 4a.

The second regression model was attempted to test whether the emotional enhancement effect in the Pe amplitude could be predicted from the N1 and P3 amplitude increases in the emotional relative to the neutral unsuccessfully inhibited trials. The predictor variables were difference measures, created by subtracting the mean N1 and P3 amplitudes recorded in the NEU UNSUCC trials from those observed in the EMO UNSUCC trials. The Pearson correlation analyses revealed that they were not significantly correlated (*r* = .33, *p* = n.s.). In turn, the predicted variable was the difference measure, created by subtracting the mean Pe amplitude recorded in the NEU response error trials from that observed in the EMO response error trials. The difference measure is an appropriate method to isolate the effect of emotion on the ERP from other effects that are not purely related to the stop-signal category. The second regression model explained 32% of the variance in the Pe amplitude difference between the EMO and NEU response conditions (*R* ^2^ = .32), *F*(2, 29) = 6.79, *p* < .01. The differential measure for the P3 amplitude was the factor that significantly accounted for variance (*ß* = .60), *t* = 3.68, *p* = .001. The differential measure for the N1 amplitude did not contribute significantly to the overall explanation of variance (*ß* = − .22), *t* = −1.37, *p* = n.s. Scatterplots and linear regression lines from the second analysis are illustrated in Fig. 4b.

### Exploratory source localization analyses

The ERPs time-locked to the stop signal in unsuccessfully inhibited trials and to the button press in erroneous response trials partly overlap in time, due to the relatively short interval between these two kinds of events. This raises the question of whether the failed-stop N1 and P3 and the erroneous-response Pe are all aggregates of stop-signal and response-monitoring activity or instead reflect functionally distinct aspects of cognitive processing and might be considered as indexes of relatively independent brain activation.^1^ To answer this question, exploratory source localization analyses were performed for the failed-stop N1, P3, and Pe, separately in the emotional and neutral conditions. The configurations of the intracranial generators giving rise to the components were estimated by using a distributed linear inverse solution, namely the low-resolution electromagnetic tomography method (LORETA; Pascual-Marqui, Michel, & Lehmann, 1994). LORETA calculates the current density at each of 2,394 voxels in the gray matter and the hippocampus of a reference brain (MNI 305 template, Brain Imaging Centre, Montreal Neurologic Institute) based on the linear, weighted sum of the scalp electric potentials. The version of LORETA applied here used a three-shell spherical head model registered to the Talairach space. The three-dimensional localization of the electrical sources contributing to the electrical scalp field was used for each participant and stop-signal condition (emotional and neutral) in unsuccessfully inhibited/erroneous-response trials. The differences in localization between conditions were computed in voxel-by-voxel *t* tests for dependent measures of the average LORETA images over the components’ time windows, based on the log-transformed power of the estimated electric current density. The analysis corresponded to a statistical nonparametric mapping (Holmes, Blair, Watson, & Ford, 1996) and relied on a bootstrap method with 5,000 randomized samples. This procedure gave the exact significance thresholds, regardless of nonnormality, and then corrected for multiple comparisons. The level of significance for all of the analyses was set to *p* < .01 for *t* values above 3.37. The coordinates of the local maxima for the statistical comparisons were listed in Table 3.

**Table 3:** Areas of statistically strongest cerebral activation for emotional as compared to neutral trials for the failed-stop N1 and P3 and the erroneous-response Pe

Component	Brain Area	BA	Coordinates^a^	*t*
N1	Bilateral paracentral lobule	4, 5, 6	– 3x, – 32y, 57z	6.48
			4x, – 32y, 57z	6.46
	Bilateral dorsal PCC/precuneus	7, 23, 31	– 3x, – 25y, 36z	6.45
			4x, – 25y, 36z	6.58
	Bilateral dorsal ACC/MCC	24	– 3x, – 18y, 43z	5.88
			4x, – 18y, 43z	6.01
	Bilateral rostral ACC/vmPFC	10, 32	– 3x, 45y, 8z	5.72
			4x, 45y, 8z	5.57
	Bilateral STG and MTG	21, 22	– 59x, – 53y, 15z	4.01
			60x, 3y, – 20z	4.27
P3	Bilateral dmPFC	9	– 10x, 38y, 22z	4.13
			11x, 38y, 22z	4.13
	Bilateral rostral ACC and dorsal ACC/MCC	24, 32	– 3x, 31y, 15z	4.08
			4x, 31y, 15z	4.08
	Right anterior insula	13	39x, – 4y, – 6z	4.17
	Right IFG	47	46x, 17y, 1z	3.72
Pe	Bilateral PCC	23, 31	– 3x, – 25y, 29z	5.41
			4x, – 25y, 29z	5.42
	Bilateral vmPFC	10	– 3x, 52y, 1z	4.83
			11x, 52y, 1z	5.10
	Bilateral rostral ACC and dorsal ACC/MCC	24, 32	– 3x, – 18y, 36z	4.80
			4x, – 18y, 36z	4.84

*ACC* anterior cingulate cortex, *BA* Brodmann area, *IFG* inferior frontal gyrus, *MCC* midcingulate cortex, *dmPFC* dorsomedial prefrontal cortex, *MTG* middle temporal gyrus, *PCC* posterior cingulate cortex, *SPL* superior parietal lobule, *STG* superior temporal gyrus, *vmPFC* ventromedial prefrontal cortex; X, Y, Z, coordinates in Talairach space, in millimeters; X corresponds to the left–right, Y to the posterior–anterior, and Z to the inferior–superior dimension. *df* = 1,31. ^a^Coordinates of local maxima.

**Fig. 5:**
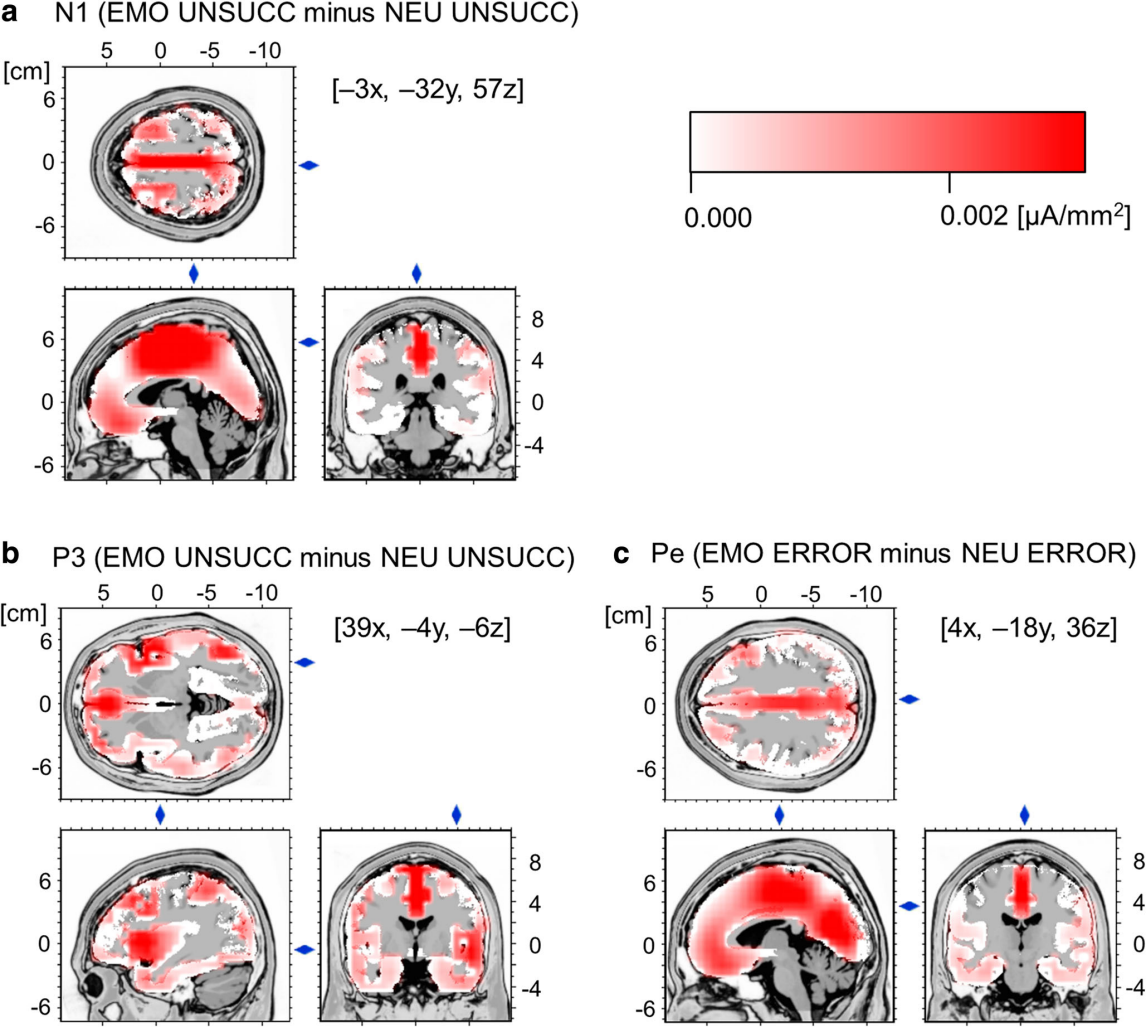
Source localization results (LORETA). **a** Direct statistical comparison between the two stop-signal conditions for the failed-stop N1 component revealed that the aversive sounds elicited significantly stronger activations than the neutral sounds within a widely distributed fronto-temporo-parietal network. **b** In turn, during the time interval corresponding to the failed-stop P3 component, emotional stop signals elicited more pronounced activations than did the neutral stop signals within the dorsomedial prefrontal cortex, anterior cingulate and midcingulate cortex, and right inferior frontal gyrus and right anterior insula (the latter difference is shown here). **c** An analogous comparison between the two erroneous response conditions for the Pe component revealed that errors committed after presentation of the aversive sounds elicited stronger activation than errors committed after presentation of the neutral sounds within the anterior cingulate, midcingulate, and posterior cingulate cortex bilaterally. The results point to the relative distinction of the intracranial generators giving rise to the emotional–neutral differences in amplitude in the failed-stop N1, P3, and Pe components. EMO = emotional stop-signal trials, ERROR = unsuccessfully inhibited responses, NEU = neutral stop-signal trials, UNSUCC = unsuccessful stop trials.

During the time interval corresponding to the N1 component (120–190 ms post-stop-signal-onset), the statistical comparison between EMO UNSUCC and NEU UNSUCC conditions showed that aversive stop signals led to stronger activation in a broad bilateral fronto-parietal cluster, encompassing the paracentral lobule [Brodmann areas (BAs) 4, 5, and 6] and midcingulate cortex (MCC) (BA 24), extending to the dorsal part of the posterior cingulate cortex (PCC) and precuneus (BAs 7, 23, 31). Furthermore, a widespread bilateral cluster with stronger activation for emotional than for neutral sounds was found within the superior temporal gyrus (STG) and middle temporal gyrus (MTG) (BAs 21 and 22). Finally, the third bilateral cluster extended from the rostral ventromedial prefrontal cortex (vmPFC) to the rostral anterior cingulate cortex (ACC) (BAs 10 and 32); see Fig. 5a.

The statistical comparison between EMO UNSUCC and NEU UNSUCC conditions within the time window of the P3 (270–400 ms post-stop-signal-onset) revealed widespread bilateral clusters with stronger activation for emotional than for neutral trials, extending from the dorsomedial prefrontal cortex (dmPFC) to the ACC and MCC (BAs 9, 24, and 32). Furthermore, a strongly right-lateralized cluster was found within the inferior frontal gyrus (IFG) and anterior insula with more pronounced activation for emotional than for neutral stop signals (BA 13 and 47); see Fig. 5b. By comparison, only very few nodes in the left IFG showed a small, and not significant, difference between the two stop-signal conditions [max at – 17x, 24y, – 20z in left BA 47; *t*(31) = 2.10].

During the time interval corresponding to the Pe component (120–270 ms post-response-onset), the statistical comparison between EMO response errors and NEU response errors showed that errors committed after presentation of the aversive stop signals led to stronger activation in broad clusters located bilaterally within the cingulate gyrus and the rostral vmPFC: one corresponding to the posterior parts of the cingulate cortex (including BAs 23 and 31), another encompassing the rostral parts of the vmPFC, and one other in the ACC and MCC (including BAs 10, 24, and 32); see Fig. 5c.

Thus, source localization analyses implied a relative alteration of the neural generators underlying the previously identified emotional–neutral difference in the failed-stop N1, P3, and Pe components, pointing to their functional distinction. This pattern of results suggests that the between-conditions difference in the failed-stop N1 and P3 amplitudes was elicited mainly by stop-signal-related processes, whereas the analogous difference in Pe amplitudes was generated by error-related processes.

### Exploratory analyses of the relationships between the SSRT and peak latency of the failed-stop P3 and Pe components

Previous research has shown that the peak or onset latency of the P3 is highly correlated with the speed of the stopping process, as measured by the SSRT (Bekker, Kenemans, Hoeksma, Talsma, & Verbaten, 2005; Wessel et al., 2016). Wessel and Aron (2015) recently proposed that the timing of the P3 is directly related to the success of response inhibition. To provide additional support for the functional distinction of the failed-stop P3 and erroneous-response Pe, exploratory analyses of the relationships between the SSRTs and latencies of these two components were performed. The peak latency was defined as the time interval between stimulus or response onsets and the maximal amplitude in the component-specific window.

The statistical comparison between the EMO UNSUCC and NEU UNSUCC conditions showed that the P3 component peaked significantly earlier in the EMO trials (*M* = 337.4 ms, *SD* = 16.0) than in the NEU trials (*M* = 368.7 ms, *SD* = 17.1), *t*(31) = 7.28, *p* < .001, *d* = 1.4. Moreover, correlational analyses revealed that the P3 latency in UNSUCC-EMO trials showed a significant correlation with the EMO SSRT (*r* = .60, *p* < .001), whereas the P3 latency in UNSUCC-NEU trials was correlated with the NEU SSRT (*r* = .38, *p* = .03). Thus, the longer P3 latency on unsuccessful stop trials corresponded with a longer SSRT in each stop-signal condition. The association between the speed of the stopping process (as measured by the SSRT) and the P3 latency was stronger in the emotional than in the neutral condition. This finding suggests that the effort to override the incorrect response activation and prevent the execution of an inappropriate action was greater within the course of an emotional than of a neutral failed stop trial. It could be hypothesized that the dynamics of action stopping, even in unsuccessfully inhibited trials, were modulated to a greater extent in response to emotional than to neutral sounds.

As in the case of the failed-stop P3, a statistical comparison between EMO and NEU response errors revealed that the Pe component peaked significantly earlier in the EMO trials (*M* = 190.5 ms, *SD* = 27.0) than in the NEU trials (*M* = 217.9 ms, *SD* = 30.3), *t*(31) = 5.44, *p* < .001, *d* = 0.9. However, contrary to the results obtained for the P3, the Pe latency in EMO response-error trials showed only a weak trend toward correlation with the EMO SSRT (*r* = – .32, *p* = .07). Even more importantly, the sign of the correlation coefficient was negative, indicating that a longer Pe latency was associated (at a level approaching significance) with a shorter SSRT in the emotional stop-signal condition. The Pe latency in NEU response-error trials was not significantly correlated with the NEU SSRT (*r* = – .25, *p* = .17). Thus, the results revealed the lack of a reliable correspondence or a very weak negative association between the Pe latency and SSRT.

To test whether the correlation coefficients of the association between the latencies of both ERP components and the SSRT per stop-signal condition differed significantly from each other, an updated version of Steiger’s *Z* test was used (Hoerger, 2013; Steiger, 1980). The analyses revealed that the EMO SSRT showed a stronger correlation with the P3 latency in UNSUCC-EMO trials than with the Pe latency in EMO response-error trials, *Z*_*H*_ = 3.68, *p* < .001. Similarly, the NEU SSRT showed a stronger correlation with the P3 latency in UNSUCC-NEU trials than with the Pe latency in NEU response-error trials, *Z*_*H*_ = 2.63, *p* < .01.

Thus, the present results suggest that the failed-stop P3 and erroneous-response Pe are differentially associated with behavioral performance measures. They also confirm that the timing of the P3 associated with action stopping may play a crucial role in the success of response inhibition. Therefore, it seems safe to conclude that the failed-stop P3 and erroneous-response Pe reflect functionally distinct aspects of cognitive control.

## Discussion

The present study had two main objectives. First, it aimed at testing whether task-relevant aversive sounds can influence task performance and lead to increased error-monitoring activity relative to a condition involving neutral sounds. Second, it was intended to show that the emotional enhancement effect on performance monitoring could be predicted from the stop-signal-related brain activity observed in the unsuccessfully inhibited trials. The behavioral and ERP data revealed that exposure to aversive stimuli improved both lower- and higher-order cognitive processes. Unpleasant, arousing sounds decreased the stop-signal reaction time and increased the inhibitory rate relative to neutral tones. These results point to an emotional facilitation effect similar to those in previously reported findings (Pawliczek et al., 2013; Pessoa et al., 2012; Senderecka, 2016).

### Perceptual processing

Aversive stop-signal trials evoked an enhanced N1 relative to neutral stop-signal trials. This observation is consistent with the results of previous reports of an increased activation of sensory areas in response to emotional sounds (Czigler et al., 2007; Grandjean et al., 2005; Plichta et al., 2011; Viinikainen, Kätsyri, & Sams, 2012; Yokosawa, Pamilo, Hirvenkari, Hari, & Pihko, 2013). A larger N1 was also registered for successful trials than for failed stop trials, which aligns with the findings of previous SST studies (Bekker et al., 2005; Dimoska & Johnstone, 2008; Hughes, Fulham, Johnston, & Michie, 2012; Lansbergen, Bocker, Bekker, & Kenemans, 2007; Senderecka, 2016).

It has been suggested that emotional stimuli selectively enhance perception and modulate attention (Pessoa et al., 2002; Pourtois et al., 2013; Vuilleumier, 2005). In the present study, aversive sounds, relative to neutral tones, generated stronger sensory representations of the stop signal (N1 component), probably leading to an enhanced attentional switch to inhibition cues. Specific forward and backward connections between the amygdala and the auditory cortex encode the emotional significance of auditory stimuli, enhance the representation of sounds in the sensory cortex and probably make them more accessible to consciousness (Mitchell & Greening, 2012). This effect indicates that discrimination between emotionally significant and insignificant stimuli occurred during early sensory stages of processing. The difference was especially pronounced in unsuccessfully inhibited stop-signal trials, in which the perceptual processing of neutral stimuli was definitely less effective than that of aversive ones.

### Inhibitory processing

Unpleasant sounds elicited a larger P3 relative to neutral tones in both successful and failed stop trials. The difference between aversive and neutral P3 amplitude was especially pronounced in the unsuccessfully inhibited trials. The P3 evoked by neutral tones in the failed stop trials was markedly attenuated. A larger P3 was also registered for successful than for failed stop trials.

The higher P3 amplitude for successfully than for unsuccessfully inhibited trials is a common result in SST studies (e.g., De Jong, Coles, Logan, & Gratton, 1990; Dimoska, Johnstone, & Barry, 2006; Dimoska, Johnstone, Barry, & Clarke, 2003; Greenhouse & Wessel, 2013; Hughes et al., 2012; Overtoom et al., 2002). According to the most influential interpretation, the successful stop P3 reflects cognitive control mechanisms, in particular the monitoring of the outcome of the inhibitory processes and their effectiveness (e.g., Nieuwenhuis et al., 2003). Consequently, the larger P3 component during successfully inhibited emotional stop-signal trials may reflect enhanced cognitive control affecting overall performance monitoring.

Various studies have pointed out that emotional stimuli are inherently motivationally salient and may capture attention automatically, in a bottom-up, reactive fashion. They may thus be considered natural targets, eliciting an increased positivity, 300–500 ms following presentation, which is similar to the P3 observed for explicitly designated targets, especially in oddball tasks (Hajcak, Weinberg, MacNamara, & Foti, 2011). This phenomenon may explain the relatively large difference between the emotional and neutral P3 amplitudes in unsuccessfully inhibited trials. The emotional P3 in failed trials consisted of a monitoring process together with an automatically occurring motivational process, which was absent in the neutral stop-signal trials. Alternatively, since the P3 component in the SST has been considered a reflection of the suppression and slowing of motor behavior (Huster et al., 2011), the relatively large P3 in emotional failed stop-trials may also signify stronger attempts to implement the correct behavior.

### Error processing

The ERN–Pe complex observed in ERPs time-locked to responses was larger for unsuccessfully inhibited than for correct responses, in line with previous research (Falkenstein et al., 1991; Nieuwenhuis et al., 2001). The ERN amplitude was comparable in the neutral and in the negative, arousing trials. This observation aligns with the findings of the previous SST study with threatening visual stimuli (Senderecka, 2016) and suggests a similar degree of postresponse conflict or mismatch between the actual response and the desired state in both stop-signal conditions (Coles et al., 2001; Falkenstein et al., 1991; Yeung et al., 2004), or a comparable increase in attentional control, regardless of stop-signal category (van Noordt et al., 2016; van Noordt et al., 2017; van Noordt et al., 2015) . The lack of modulation of the ERN might also indicate that the subjective significance or aversiveness of an error was similar for both sound categories, at least at this early stage of response monitoring (Gehring et al., 1993; Hajcak & Foti, 2008; Hajcak et al., 2005; Inzlicht & Al-Khindi, 2012; Schmeichel & Inzlicht, 2013). This result stands in contrast to previous reports that have found that short-duration affective states influence the size of the ERN (Larson et al., 2006; Ogawa et al., 2011; Pfabigan et al., 2013; Riesel et al., 2012; Wiswede et al., 2009). However, it is in line with the less numerous, although informative, studies that have failed to observe ERN amplitude variation in response to affective state induction (Moser et al., 2005; Olvet & Hajcak, 2012; Paul et al., 2017). It should be noted that comparing the present results with those of previously published studies is difficult, because of the different natures of the tasks (flanker task, Stroop task, go/no-go task, stop-signal task), different natures of the errors (hand errors in choice-reaction tasks in flanker vs. inhibition errors in go/no-go and stop-signal task), and finally the different natures of the affective-state inductions (based on bottom-up influence of briefly presented task-irrelevant or -relevant visual or auditory stimuli vs. more abstract top-down emotional manipulation). The diversity of these results indicates that short-duration affective states can produce different effects during the early stages of error monitoring, depending on specific procedure demands, and certainly points to the need for further research.

An important finding of this study is that the second component associated with error processing was significantly greater in the emotional than in the neutral trials. Traditionally, the Pe has been considered to be a conscious evaluation of an error, or affective processing related to an erroneous response (see Overbeek et al., 2005, for a review). More recently, Steinhauser and Yeung (2010) proposed that the Pe reflects the accumulation of evidence that an error has occurred. The results of the present investigation indicate that this second aspect of error processing was enhanced in the emotional condition, suggesting that short-duration affective states, induced by aversive, arousing sounds, exert a positive influence on error monitoring. It seems reasonable to suppose, in accordance with the results of the previous study (Senderecka, 2016), that the Pe emotional effect reflects an increase in the error significance or an enhancement of the error evidence strength after the presentation of the aversive stop signal.

### Emotional enhancement of error detection and its neural mechanisms

To explore associations between the two ERP components time-locked to the stop signal (N1, P3) and the Pe component time-locked to the erroneous response, two multiple regression analyses were performed. The first analysis was conducted regardless of the category of the stop signal. It revealed that both selected factors—the N1 and P3 amplitudes observed in the unsuccessfully inhibited trials—significantly accounted for the Pe component variance, explaining its large amount (66%). The greater Pe amplitude was associated, in general, with a larger N1 and P3 in unsuccessfully inhibited trials. This suggests that error processing was stronger if the erroneous response directly followed the stop signal, which was effectively processed on the perceptual and cognitive control levels. The second regression model was focused on difference measures (emotional minus neutral) and revealed that the emotional increase of the P3 amplitude was the only factor that significantly accounted for the emotional enhancement effect in the Pe amplitude. The differential measure of the N1 amplitude did not contribute significantly to the overall explanation of the effect.

The auditory N1 is thought to consist of a complex of at least three separate subcomponents that are generated in the temporal (auditory cortex), as well as parietal (association cortex) and frontal (motor and premotor cortices) lobes (Näätänen & Picton, 1987). In the present study, the failed-stop N1 emotion effect was indeed expressed as stronger activation in the largely distributed temporo-fronto-parietal network, which closely correspond to findings reported in previous research (Bröckelmann et al., 2011).The activation of such a broad array of neural circuitry has been commonly observed in neuroimaging studies on selective directed attention. The network has been implicated as underlying the control of auditory and visual attention, and modulating processes driven by current goals, task relevance, or inherent stimulus salience (Bidet-Caulet & Bertrand, 2005; Corbetta & Shulman, 2002; Fritz, Elhilali, David, & Shamma, 2007). Thus, the present results suggest that the greater salience of the aversive stop signals was probably the factor that has led to the stronger engagement of the multisensory attention network during emotional stop-signal condition (Vuilleumier, 2005). Additionally, it could be hypothesized that the connection between sensory areas and amygdala was regulated by top-down signals from vmPFC (Vuilleumier, 2009). The overall association of the N1-Pe amplitudes (when collapsed across stop-signal conditions) points to the possibility that the earlier activation of the temporo-fronto-parietal attention network can influence to some degree the error-monitoring system. However, the results of the second regression analysis revealed that the emotional–neutral difference, observed at the sensory stage of sounds processing, was not crucial for the subsequent emotional enhancement effect on error detection.

In the failed-stop P3 time range the aversive sounds elicited significantly stronger activation than the neutral sounds within the right IFG, right anterior insula, bilateral dmPFC, and ACC/MCC, which is in agreement with the notion that P3 is generated by multiple neuronal sources (Nieuwenhuis, Aston-Jones, & Cohen, 2005; Polich, 2007). The right IFG constitutes the key node of the inhibitory neural network, whose activation is consistently observed in neuroimaging studies on stop-signal performance (Aron, Fletcher, Bullmore, Sahakian, & Robbins, 2003; Aron & Poldrack, 2006; Hughes et al., 2014; Hughes et al., 2012; Hughes, Johnston, Fulham, Budd, & Michie, 2013). Its engagement has been implicated as critical for inhibiting an already initiated manual response (for reviews, see Aron, 2011; Aron, Robbins, & Poldrack, 2004, 2014). The activation of the insula (especially its anterior part) is also supposed to contribute to inhibitory control, such as response suppression or slowing (Aron & Poldrack, 2006; Hughes et al., 2013; Huster et al., 2011) or at least to reflect autonomic arousal related to stopping (Ramautar, Slagter, Kok, & Ridderinkhof, 2006). The neuroimaging data also suggest that the right anterior insula activation varies with stop-signal task difficulty (Hughes et al., 2013).

The dmPFC has been reported in many neuroimaging studies to be generally sensitive to salience, novelty, and other potentially relevant features (including aversiveness) of the presented auditory and visual stimuli, although the exact reported coordinates differ between experiments (Dien, Spencer, & Donchin, 2003; Friedman, Cycowicz, & Gaeta, 2001; Hermans, Henckens, Roelofs, & Fernández, 2012; Huang, Belliveau, Tengshe, & Ahveninen, 2012; Kiehl, Laurens, Duty, Forster, & Liddle, 2001; Kiehl et al., 2005). Thus, the mechanism standing behind the differentiated dmPFC activation in aversive and neutral conditions in the P3 time window seems to operate at least to some extent in a bottom-up fashion. In addition, the activation of the dmPFC (viz. BA 9) was also observed in neuroimaging research during response suppression (Menon, Adleman, White, Glover, & Reiss, 2001). Similarly, both the ACC (Hughes et al., 2014) and the MCC (Huster et al., 2011) were identified as important nodes in the neural network supporting motor inhibition during SST performance.

In the Pe latency range emotion effect was expressed as stronger activation in the largely distributed network, encompassing anterior cingulate, midcingulate and posterior cingulate cortex. The contribution of these medial brain areas to the generation of the Pe component has been previously revealed in numerous studies using dipole modeling or LORETA (Herrmann, Römmler, Ehlis, Heidrich, & Fallgatter, 2004; Mathewson, Dywan, & Segalowitz, 2005; O’Connell et al., 2007; Paul et al., 2017; van Boxtel, van der Molen, & Jennings, 2005; van Veen & Carter; 2002; Vocat, Pourtois, & Vuilleumier, 2008). These results confirm that the cingulate cortex is broadly responsive to the outcomes of actions and largely involved in evaluating performance.

The summary of the areas showing stronger activation for aversive than for neutral trials indicates that both the P3 and Pe emotional effects had in common at least one putative source, corresponding to the anterior cingulate/midcingulate cortex. Hence, it seems reasonable to tentatively assume that this convergence may point to a neural mechanism underlying the P3–Pe amplitude correlation. Interestingly, in an SST study, Huster et al. (2011) examined the association of performance-monitoring and inhibition-related ERPs and BOLD responses by means of EEG-informed analysis of fMRI data. The results revealed that both the stop-signal-related P3 and the ERN/Pe were correlated with the time courses of an activity localized predominantly in the anterior regions of the MCC, and additionally in the pre-supplementary motor area (preSMA), the anterior insula, the putamen and the globus pallidus. Thus, it has been suggested that the stop-signal-related P3 and ERN/Pe complex rely at least to some degree on a similar neural network and may both reflect activity changes within the anterior MCC and its connected regions.

This raised the question: How is the MCC influenced by affective stimuli so that they can lead to more effective performance monitoring? Studies on monkeys have suggested that the MCC receives widespread direct and indirect inputs from emotion-related brain regions, including signals from the orbitofrontal cortex and insula (Morecraft & van Hoesen, 1998). A number of neuroimaging studies (e.g., Morrison, Peelen, & Downing, 2007; Pereira et al., 2010) have indeed shown that the midcingulate responses are modulated by negatively valenced stimuli. Importantly, Pereira and colleagues hypothesized that the MCC likely plays a crucial role in the implementation of defensive, “freezing”-like behaviors, involving the integration of negatively valenced and motor information. Following this line of interpretation, it can be assumed that, in the present study, the MCC was receiving affective information from emotion-related regions during aversive contexts, and was sending it to other parts of the motor and performance monitoring system network, leading either to successful response inhibition or at least to increased erroneous response processing on failed stop trials.

### Conclusions and future directions

This study investigated, first, whether task-relevant, unpleasant, arousing sounds can modulate task performance and lead to increased error-monitoring activity relative to a neutral task condition, and second, whether the emotional enhancement effect on performance monitoring could be predicted from the stop-signal-related brain activity observed in the unsuccessfully inhibited trials. The results revealed that aversive stimuli facilitated inhibitory processing by decreasing the stop-signal reaction time and increasing the inhibitory rate relative to neutral tones. The perceptual processing of affectively significant stop signals resulted in a stronger N1 auditory component. Unpleasant sounds also evoked a larger P3 relative to neutral tones in both successful and failed stop trials, indicating an enhancement in cognitive control operations. The early stage of error processing was similar in the emotional and neutral trials, as indexed by the ERN amplitude. However, the Pe component, which is associated with the conscious evaluation of an error, affective processing related to an erroneous response or the accumulation of evidence that an error has occurred, was markedly larger in the emotional than in the neutral condition.

Both stop-signal-related states examined in the present study—namely perceptual processing of the stop signal and inhibition monitoring—influenced conscious error detection, indexed by the late positivity of the response-locked event-related brain potential. This suggests that error processing was stronger if the erroneous response directly followed the stop signal, which was effectively processed on the perceptual and action monitoring levels. However, the only factor that accounted for the difference in error detection between the emotional and neutral context was inhibitory performance monitoring. Large emotional enhancement of the P3 amplitude was associated with an increase of error significance in failed, aversive stop trials. In other words, the cognitive system found more inhibition-monitoring evidence to effectively detect errors on aversive, unsuccessfully inhibited trials than on neutral ones. This observation seems to point to the crucial role of the MCC in the execution of internal processes leading to the emotional enhancement of error detection. Since the MCC constitutes a node where information about affect and the need for control are linked to motor centers (Shackman et al., 2011), this frontal area is probably responsible for executing goal-directed behavior and simultaneously optimizing performance in response to emotional cues. The results of the present study provide further support for the notion that Pe amplitude can be predicted from the brain activity that occurs even before error commission.

Some limitations and future directions of the present work should be mentioned here. First, although LORETA is an empirically well supported and widely used source localization method (Pascual-Marqui, Esslen, Kochi, & Lehmann, 2002; Pascual-Marqui et al., 1994), the inverse solution results obtained in the present study should be interpreted with caution, because they necessarily remain imprecise as a mathematical reconstruction.

Second, in the present study the affective significance of stop signals was manipulated; however, the aversive and neutral valence categories were not matched for arousal level, which is very difficult to ensure in the case of short, auditory stimuli. For this reason it remains unclear whether emotion-modulated response inhibition is related to valence (aversive – neutral) or to arousal (arousing – neutral), two affective dimensions that are widely considered to explain the variance in emotional meaning (Lang, Greenwald, Bradley, & Hamm, 1993).

Third, according to several models, unpleasant stimuli elicit more rapid or more prominent affective responses, involving cognitive and physiological changes, than pleasant stimuli (Cacioppo & Gardner, 1999). The existence of such a negativity bias has received experimental support from numerous studies on brain activity (e.g., Ito, Larsen, Smith, & Cacioppo, 1998). For this reason, inhibitory performance was compared across two stop-signal conditions: aversive and neutral. However, it would also be worthwhile to replicate the present results using positively valenced sounds.

Fourth, recent studies have suggested that much of the top-down control in response inhibition tasks takes place before the inhibition signal is presented (Elchlepp, Lavric, Chambers, & Verbruggen, 2016; Langford, Krebs, Talsma, Woldorff, & Boehler, 2016a; Langford, Schevernels, & Boehler, 2016b; for a theoretical account, see Verbruggen, 2016). An analysis of the ERPs for go stimuli revealed that proactive inhibitory control may bias stimulus detection, action selection, and action execution in the SST. Thus, further research will surely be needed to determine whether error-monitoring efficiency could also be predicted from go-related brain activity, even when it occurred several hundred milliseconds before stop-signal presentation and error commission.

## Footnotes

1 I thank the reviewers for drawing this issue to my attention.
